# The Effect of Compost, Host Resistance, and Chemical Treatment Interaction on Complex Wilt Disease Management on Hot Pepper (*Capsicum annuum* L.) in Jabi Tehena District, Northwestern Ethiopia

**DOI:** 10.1155/tswj/3626221

**Published:** 2025-07-15

**Authors:** Mastewal Alehegn, Chemeda Fininsa, Habtamu Terefe, Mashilla Dejene, Wassu Mohammed

**Affiliations:** ^1^Department of Horticulture, Debre Markos University, Debre Markos, Ethiopia; ^2^School of Plant Sciences, Haramaya University, Dire Dawa, Ethiopia

**Keywords:** AUDPC, chemical, disease severity, epidemics, host resistance, yield loss

## Abstract

Hot pepper is a vital vegetable crop traditionally valued for its commercial importance and role in rural economies, with its fruits consumed fresh, dried, processed, or used as condiments. Hot pepper wilt, a complex disease caused by various soilborne pathogens, significantly influenced hot pepper crops. This study is aimed at evaluating the combined effects of compost application, host resistance, and chemical treatments on seeds and seedlings in managing this disease, as well as its effect on yield. Eighteen treatment combinations were tested in a split–split plot design with three replications. The findings showed highly significant differences (*p* ≤ 0.001) in disease severity, area under disease progress curve (AUDPC), and agronomic traits across the treatment combinations. The study found that the Melka Zala variety, when treated with Apron Star and transplanted into compost-treated plots, exhibited the lowest disease severity (23%), AUDPC (478.33%-days), and disease progress rate (0.0034 units/day). In contrast, the Mareko Fana variety, grown in compost-untreated and control plots, showed the highest disease severity (54%), AUDPC (1426.67%-days), and disease progress rate (0.0114 units/day). Additionally, Melka Zala yielded the highest marketable fruit yield (2.42 t ha^−1^) and total fruit yield (2.47 t ha^−1^) when the seeds and seedlings were treated with Apron Star Fungicide and transplanted into compost-treated plots. Treating Melka Zala seeds and seedlings with Apron Star fungicide and transplanting them into compost-treated plots resulted in twice the net benefit and marketable fruit yield compared to other treatment combinations. In conclusion, using the Melka Zala variety treated with Apron Star and grown in compost-treated plots effectively controlled the disease and improved yield, suggesting this approach as a viable strategy for farmers in the study area and similar agroecological zones to manage wilt disease.

## 1. Introduction

Hot pepper, belonging to the Solanaceae family, is a self-pollinating, diploid (2*n* = 24) crop closely related to tobacco, petunia, tomato, and potato [1]. Globally, it is a vital vegetable crop due to its commercial value and significance in rural economies [2, 3]. Typically grown as an annual crop, hot pepper holds substantial economic importance worldwide [4]. In Ethiopia, it is a favored vegetable cultivated during both rainfed and irrigation seasons, contributing to smallholder farmers' incomes and export revenue [5]. The fruits, consumed fresh, dried, or processed, are also used as condiments [6]. Rich in vitamins A and C, hot pepper also provides vitamin B2, potassium, phosphorus, and calcium. Additionally, it is utilized in treating arthritis, asthma, coughs, the common cold, and infected wounds [7, 8]. It serves as a significant cash crop in the study area, meeting local demand and offering export potential.

Globally, hot pepper production is dominated by countries such as China, India, Mexico, and Turkey, which collectively hold a substantial share of the world's output. According to the FAO (2021) report, China leads in production, contributing over 50% of the global supply. Ethiopia also maintains a notable position in the global hot pepper production landscape [9]. While hot pepper is cultivated in various parts of Ethiopia, the primary producing regions are the Amhara, Oromia, and Southern Nations, Nationalities, and Peoples' Regional States (SNNPRS) [10]. During the 2020/2021 cropping season, Ethiopia produced 0.30 million tons of hot pepper on approximately 0.17 million hectares, achieving a productivity rate of 1.76 t ha^−1^. In the Amhara region, specifically, hot pepper was grown on about 0.07 million hectares, yielding 0.17 million tons with a productivity rate of 1.86 t ha^−1^ [11]. Ethiopia's hot pepper production, while significant in total area cultivated, lags behind leading global producers such as China and India in both volume and productivity per hectare [9].

Despite its significant importance, hot pepper production faces numerous challenges, including the use of inferior varieties, poor cultural practices, and a rising incidence of fungal, bacterial, and viral diseases [12, 13]. Its production is significantly constrained by wilt complex disease, a major threat in many growing regions, including Ethiopia. This disease is caused by *Rhizoctonia* spp., *Fusarium* spp., *Verticillium* spp., *Sclerotinia* spp., *Pythium* spp., and *Phytophthora* spp. complexes and is particularly problematic on a global scale [14]. They produce survival structures such as chlamydospores or microsclerotia that remain dormant until favorable conditions return. This disease can impact plants at any growth stage by causing wilt syndrome [15]. Yield losses due to the disease vary based on cultivar, soil type, climate, cropping practices, and pathogen strain [16]. Wilt pathogens such as *Rhizoctonia* spp., *Fusarium* spp., *Verticillium* spp., *Sclerotinia* spp., *Pythium* spp., and *Phytophthora* spp. can lead to yield losses of 50%–75% in crops like wheat, cotton, maize, vegetables, fruits, and ornamentals [17]. In northwestern Ethiopia, hot pepper wilt complex disease currently poses a significant challenge to both the quality and quantity of hot pepper production, causing considerable yield losses [18, 19]. The complex interaction among these pathogens complicates diagnosis and management, making it a persistent challenge for farmers and researchers alike [20, 21]. Hot pepper wilt complex diseases significantly reduce marketable yield by stunting plant growth, leading to fewer and smaller fruits. They also degrade fruit quality with symptoms like discoloration and deformities, lowering marketability and potentially reducing prices or causing rejection by buyers. Additionally, some pathogens can cause premature plant death or severe damage before fruits fully mature [22]. Growing hot pepper cultivars resistant to fungal and bacterial wilt complex is the most promising, effective, economical, and environmentally friendly approach to manage the disease [23]. Organic matter is used as an alternative approach for suppressing fungal and bacterial wilt diseases by enhancing beneficial microorganisms and improving the physical, chemical, and biological properties of the soil [24]. Soil amendment is an alternative choice to manage bacterial wilt and root rot diseases in the major solanaceous crop-growing regions of Ethiopia [25, 26]. *Fusarium* wilt disease incidence is reduced by up to 80% when organic matter is applied to the soil [27]. Apart from disease management, soil amendment enhances plant growth and soil fertility and increases beneficial soil microorganisms [28]. Likewise, fungicides are used effectively for the management of plant diseases since the compounds have a direct effect on pathogen growth and reproduction [29]. Fungicidal seed treatment can prevent seed deterioration and seedling blight caused by soilborne pathogens by killing or inhibiting seedborne pathogens and creating a protective zone around seeds [30]. Integrated use of soil amendments, biocontrol agents, and judicious pesticide use is regarded as an effective approach to managing wilt complex disease [31]. Furthermore, the wilt complex disease of hot pepper is well managed through an integrated approach involving cultural, biological, and chemical applications [32, 33]. This comprehensive approach not only targets diverse aspects of disease prevention but also aims at reducing reliance on single control measures, thereby promoting long-term management effectiveness and environmental stewardship.

Initial studies that combine multiple strategies for managing the disease indicate the need for further research to ensure sustainable hot pepper production [22]. The key principle of integrated disease management is to use multiple strategies together, which may have additive or synergistic effects. The synergistic effect in disease management implies that the combined use of multiple control measures or strategies results in a more effective outcome than the sum of their individual effects [34]. Overall, this underscores the importance of adopting integrated and multifaceted strategies in disease management to achieve more effective, sustainable, and resilient crop production systems [35]. However, no field-level research has been conducted on these integrated schemes to manage hot pepper wilt complex disease in the study area. Moreover, despite its economic and nutritional importance, integrated management strategies for wilt complex remain limited, demanding urgent research and intervention. Therefore, it is hypothesized that combining multiple management strategies could decelerate the progress of this disease. To address these research gaps and hypotheses, the current study is aimed at evaluating the effects of integrating compost application, host resistance, and chemical seed and seedling treatments on the development of wilt complex disease and yield performance.

## 2. Materials and Methods

### 2.1. Experimental Site Description

**Figure 1:**
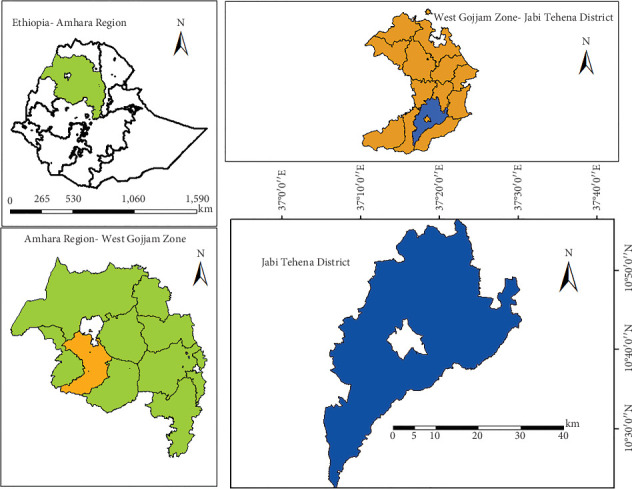
Mapping and figure of the study area.

A field experiment was conducted at the Geraye Farmers' Training Center (Geraye FTC) in Jabi Tehena District, northwestern Ethiopia, during the main rainy seasons of 2020 and 2021 in a naturally infected area. The Geraye FTC is situated at 10°42⁣′ N latitude and 37°16⁣′ E longitude, at an elevation of 1917 m above sea level (Figure 1). The area received an average total rainfall of 1099.4 mm in 2020 and 937 mm in 2021 during the cropping periods from May to October. The mean minimum and maximum temperatures were 12.86°C and 25.91°C in 2020 and 12.22°C and 23.92°C in 2021. The relative humidity ranged from 64% to 92% in 2020 and from 58% to 91% in 2021 during the main cropping seasons. The soil type at the site is characterized as red nitisol according to the National Meteorological Agency Data of the District's 2020 and 2021 cropping seasons.

### 2.2. Seed Source, Seedbed Preparation, and Seedling Treatment

Hot pepper seeds from the Melkasa Agricultural Research Center (MARC) were used in the study. Each variety's seeds were treated with Apron Star and Cupricide chemicals at sowing and during transplanting at a rate of 2 g (active ingredient) per kilogram of seed, following Zewdie [36] (Table 1). These treated and untreated seeds were then planted in adjacent 1 × 1 m plots in a nursery. Planting took place on May 25, 2020, and May 26, 2021, during the rainy seasons. The seeds were lightly covered with fine soil and mulched with dry grass until germination. After 52 days in 2020 and 55 days in 2021, the seedlings were relocated from the nursery, treated with each chemical for 3 min for coating, and then transplanted into the field on July 15, 2020, and July 19, 2021, respectively. Weeding and cultivation were carried out as needed, as recommended by Getahun and Habtie [6].

### 2.3. Treatments, Experimental Design, and Management

The study incorporated 18 treatment combinations, including two compost application levels (compost-treated and compost-untreated), three hot pepper varieties (Melka Awaze, Melka Zala, and Mareko Fana) (Table 2), and two types of seed and seedling treatment chemicals (Apron Star 42% WS and Cupricide 77% WP), along with a control group. Melka Awaze and Melka Zala varieties are resistant to soilborne diseases, while Mareko Fana is susceptible to wilt disease [39]. The two fungicides have different modes of action and have distinct chemical compositions (Table 1). The compost, made from materials such as manure, crop residues, and kitchen waste from local gardens, was thoroughly decomposed and matured over 2 months by turning it every 15 days. It was applied uniformly at a rate of 10 t ha^−1^ in the field a month before transplanting the seedlings, in accordance with established guidelines [40, 41].

The field investigation was set up using a split–split plot design with three replications. The main plots were given compost at two levels, the subplots had three varieties of hot peppers, and the sub-subplots were treated with two types of chemicals along with a control. The main plot was 14.4 m long and 8.4 m wide, totaling 120.96 m^2^. Subplots were 2.8 m wide and 7.2 m long, totaling 20.16 m^2^, while sub-subplots were 2.8 m wide and 2.4 m long, totaling 6.72 m^2^. Each plot had four rows with eight plants per row, making 32 plants per plot. Block spacing was 1 m, and spacing between plots, rows, and plants was 0.8, 0.7, and 0.3 m, respectively. Standard agronomic practices like weeding and cultivation were carried out as per recommendations.

### 2.4. Data Collection

#### 2.4.1. Disease Data

Disease severity was monitored five times at 10-day intervals, starting 45 days after transplanting in both main rainy seasons (2020 and 2021). To assess disease severity, 10 randomly selected plants from the central two rows of each plot were tagged before wilt symptoms appeared. The severity was evaluated using a scale from 0 to 5 [42], where 0 = *no visible infection and healthy plants*, 1 = *slight leaf yellowing*, 2 = *old lower leaf yellowing and plant wilting*, 3 = *lower leaves shedding and stunted plants*, 4 = *all leaves shedding with stem collapse and some plant deaths*, and 5 = *total plant death*. These ratings were then converted into a percentage severity index (PSI) using the formula proposed by Wheeler [43].

(1) $$\begin{array}{c} \text{PSI}=\frac{\text{The sum of numerical ratings }}{\text{The total number of plants scored}\times \text{maximum score on a scale}}\times 100. \end{array}$$

The area under disease progress curves (AUDPCs) in percent-days was calculated [44] from disease severity data using the formula:

(2) $$\begin{array}{c} \text{AUDPC}=\overset{n-1}{\underset{i=1}{\sum }}\left(\frac{\;x_{\text{i}}+x_{\text{i}+1}}{2}\right)\left(t_{\text{i}+1}-t_{\text{i}}\right), \end{array}$$

where *x*_*i*_ is the cumulative disease severity expressed as a proportion at the *i*^th^ observation, *t*_*i*_ is the time of the *i*^th^ assessment in days from the first assessment, and *n* is the total number of observations/assessments. AUDPC was expressed in percent-days, as severity was recorded in percent and time in days.

To calculate the disease progress rate, data on disease severity were utilized. Since hot pepper wilt complex disease follows a monocyclic reproduction pattern, a monomolecular epidemiological model [ln(1/1–*y*)] was selected. This model was applied to estimate the disease progress rate by performing linear regression on transformed severity data against days after seedling transplantation using the formula detailed by Madden et al. [45].

 $$\begin{array}{c} y_{t}=1-\left(1-y_{o}\right)e^{-r_{M}t}, \end{array}$$

where *y*_*t*_ is the percentage of severity at the *t*^th^ assessment date, *y*_*o*_ is the percentage of initial severity at the *t*^th^ assessment date, *t*_*i*_ is the time of the *i*^th^ assessment in days from the first assessment date, and *r*_*M*_ is the rate parameter determined by the production of inoculum by infected individuals/lesions per unit area of diseased tissue.

#### 2.4.2. Growth, Yield, and Yield Components

Growth, yield, and yield component parameters were assessed and recorded from each experimental plot. These include seedling survival percentage, plant height, number of fruits per plant, fruit diameter, fruit length, marketable fruit yield, unmarketable fruit yield, and total fruit yield. Plant height was measured using a meter on 10 pretagged plants in the two central rows at physiological maturity. The number of fruits per plant was counted from two harvestable rows, with 10 randomly pretagged plants assessed at the time of harvest. Fruit length and diameter were also measured using a vernier caliper on 10 marketable fruits per plot at harvest, and mean values were calculated. Seedling survival percentage and stand count percentage were calculated as the total number of plants found relative to the total plants transplanted 2 weeks after transplanting and during the harvest period, respectively. Marketable fruit yield was determined from two central harvestable rows by sorting dried fruit based on color, shape, shininess, and size (length and width ranging from 6 to 12 cm and 1 to 3 cm, respectively); expressed in kg/ha^−1^; and then converted to kg/ha^−1^ [9]. Unmarketable fruit yield was determined by sorting diseased, discolored, shrunken, and small-sized fruit from marketable dried fruits, also expressed in kilograms per hectare, and then converted to t/ha^−1^. Unmarketable hot pepper fruit yield was determined by fruits that do not meet specific market standards for size, shape, color, and overall appearance, typically falling outside the size range of 6–12 cm in length and 1–3 cm in width [9]. Total fruit yield was the combined marketable and unmarketable fruit yields from each of the two central harvestable rows, expressed in kg/ha^−1^ and converted to kg/ha^−1^. These methods were in accordance with Getahun and Habtie [6] and Daniel and Abrham [10].

### 2.5. Relative Yield Loss

Yield losses were evaluated to understand how treatment combinations affected hot pepper fruit yield performance. The relative yield loss for each treatment was determined using the formula outlined by Robert and James [46], which calculates the percentage reduction in yield compared to the maximum yield achieved in protected plots.

(3) $$\begin{array}{c} \text{RYL }\left(\%\right)=\frac{\text{YP}-\text{YUP }}{\text{YP}}\times 100, \end{array}$$

where RYL is the relative yield loss (reduction of the yield), YP is the mean yield which was obtained from plots with maximum protection, and YUP is the mean yield which was obtained from plots with minimum protection.

### 2.6. Data Analysis

Hot pepper disease severity, AUDPC, yield, and yield components for each treatment combination underwent analysis of variance (ANOVA) using the PROC GLM procedure of SAS Version 9.4 [47] to assess the impact of integrated management on wilt complex disease and crop yield. Mean differences were determined using the least significant difference (LSD) at a 5% significance level. Compost, varieties, and chemicals were treated as fixed factors in the model, while year and replication were considered random factors.

Pearson's correlation matrix was utilized in SAS Version 9.4 [47] to examine associations among the parameters. Data from both years were combined for analysis since they exhibited homogeneous variances, confirmed by Bartlett's test and a nonsignificant *F*-test [48]. Additionally, economic feasibility analysis was conducted following CIMMYT's [49] guidelines. This analysis included total input cost (fixed and variable), gross benefit, net benefit, and benefit–cost ratio to assess economic viability.

## 3. Results

### 3.1. Disease Severity

The combined ANOVA over years revealed statistically significant differences (*p* ≤ 0.05) in disease severity and AUDPC at the final assessment dates (85 DAT). However, there were no significant differences (*p* ≤ 0.05) in mean disease severity and AUDPC values when considering interactions between cropping seasons and compost-treated varieties and chemicals (Table 3). For instance, relatively lower initial (5.33%) and final (23%) disease severities were observed when using Melka Zala variety seeds and seedlings treated with Apron Star and then transplanted into compost-treated plots. Similarly, the second-lowest (27.33%) final disease severity was noted when Melka Zala seedlings were transplanted into compost-treated plots and treated with Cupricide (Table 4).

Conversely, the highest initial (18.67%) and final (54.0%) disease severities were observed when the Mareko Fana variety was transplanted into compost-untreated plots without any chemical treatment for its seeds and seedlings (Table 4). In contrast, statistically significant differences (*p* ≤ 0.05) in final disease severity were noted when Melka Awaze variety seeds and seedlings were treated with Apron Star and Cupricide before being transplanted into compost-untreated plots (Table 3). Lower disease severity and better wilt control were achieved when Melka Zala variety seeds and seedlings were treated with Apron Star and transplanted into compost-treated plots. Similarly, there were no statistically significant differences (*p* ≤ 0.05) in disease severity between control plots and those where the Melka Awaze variety was transplanted into compost-treated plots, regardless of whether the seeds and seedlings were treated with Apron Star or Cupricide. Overall, transplanting the Melka Zala variety into compost-treated plots and treating its seeds and seedlings with Apron Star reduced initial disease severity by 66.82% and final disease severity by 57.41% compared to the Mareko Fana variety in compost-untreated control plots (Table 4).

### 3.2. AUDPC

The pooled ANOVA results indicated highly significant differences (*p* ≤ 0.001) in AUDPC among various treatment combinations (Table 4). The results revealed that the highest AUDPC value (1426.67%-days) was observed in the Mareko Fana variety when transplanted into compost-untreated and control plots, while the lowest AUDPC value (478.33%-days) was seen in the Melka Zala variety when transplanted into compost-treated plots and treated with Apron Star. No significant differences (*p* ≤ 0.05) were found in AUDPC values when comparing the Melka Zala variety transplanted into compost-treated plots and treated with Cupricide against those treated with Apron Star and transplanted into compost-untreated plots (Table 4). Melka Zala transplanted into compost-treated plots and treated with Apron Star showed a 22.22% reduction in AUDPC compared to Melka Awaze in similar conditions treated with Cupricide. Similarly, there was a 13.40% AUDPC reduction in the Melka Zala variety transplanted into compost-treated plots compared to the Melka Awaze variety treated with Apron Star. Consistently lower AUDPC values were observed when the Melka Zala seeds and seedlings were treated with Apron Star and transplanted into compost-treated plots. This combined approach of using compost, the Melka Zala variety, and Apron Star demonstrated reduced AUDPC values compared to Mareko Fana transplanted into compost-untreated and control plots (Table 4).

### 3.3. Disease Progress Curve

**Figure 2:**
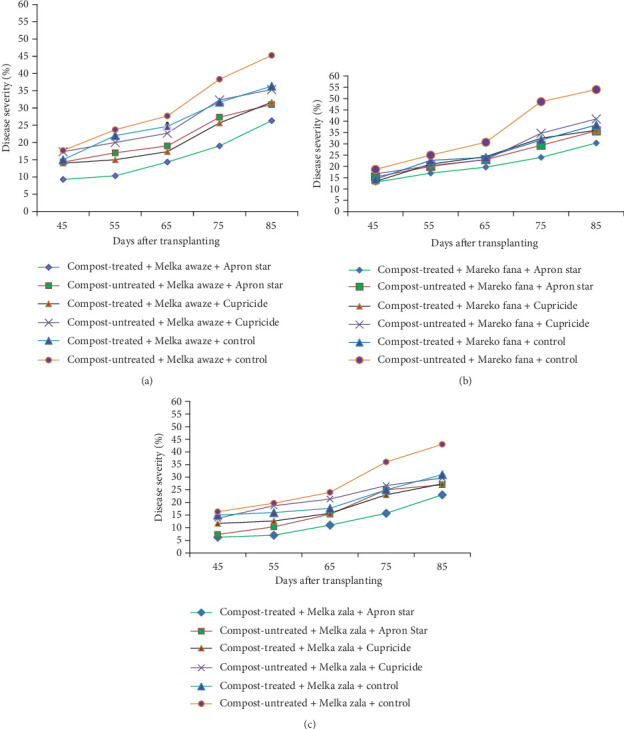
Hot pepper wilt complex disease progression curves as affected by integrated use of compost and chemical treatment, along with the control on (a) Melka Awaze, (b) Mareko Fana, and (c) Melka Zala varieties in the Jabi Tehena District during the 2020 and 2021 main rainy seasons.

A crucial aspect of summarizing a plant disease outbreak and its influencing factors is to track disease levels at various points during the growing seasons. In this study, disease progress curves were generated using mean disease severity values of hot pepper wilt complex disease. These curves depicted how disease developed over time and showcased the impact of treatments on epidemic development. Figures 2a, 2b, and 2c illustrate the disease progress curves for different hot pepper varieties when their seeds and seedlings were treated with chemicals and transplanted into either compost-treated or compost-untreated plots.

The disease progress curves revealed variations in disease levels among hot pepper varieties under different treatment combinations. During the disease assessment period, Melka Awaze, Mareko Fana, and Melka Zala varieties treated with Apron Star showed slower epidemic development compared to those treated with Cupricide or the control, regardless of whether seedlings were transplanted into compost-treated or untreated plots (Figures 2a, 2b, and 2c). Notably, the disease progress curve was slower for the Melka Zala variety when its seeds and seedlings were treated with Apron Star and transplanted into compost-treated plots across different disease assessment days (Figure 2c). Conversely, the highest disease progress curve was observed for the Mareko Fana variety when its seedlings were transplanted into compost-untreated plots without any chemical treatment (Figure 2b).

### 3.4. Disease Progress Rate

Table 5 presents the hot pepper wilt complex disease progress rate and parameter estimate (intercept). The disease progress rate varied depending on the treatment combinations received by each variety. The Mareko Fana variety exhibited the fastest disease progress rate (0.0114-unit day^−1^) when its seedlings were transplanted into compost-untreated and control plots. Following closely, the Melka Awaze variety showed the second-fastest disease progress rate (0.0091-unit day^−1^) under similar conditions (Table 5). Conversely, the Melka Zala variety displayed a slower disease progress rate (0.0034-unit day^−1^) when its seeds and seedlings were treated with Apron Star and transplanted into compost-treated plots. Generally, the hot pepper wilt complex disease progress rate was notably slower when hot pepper varieties' seeds and seedlings were treated with Apron Star and transplanted into compost-treated plots compared to other treatment combinations.

### 3.5. Hot Pepper Yield and Yield Components

#### 3.5.1. Growth Parameters

The application of compost and various chemicals significantly impacted the mean growth, yield, and yield components of hot pepper varieties compared to plots without compost application or chemical treatments (Table 6). Specifically, the Melka Zala variety showed significantly higher seedling survival (90.63%) with Apron Star treatment in compost-treated plots, while the Mareko Fana variety exhibited lower seedling survival (73.44%) in compost-untreated and control plots (Table 6). Additionally, Melka Awaze variety seeds and seedlings treated with Apron Star and transplanted into compost-treated plots resulted in the tallest plants (82.97 cm) compared to the Mareko Fana variety transplanted into compost-untreated and control plots, which had a plant height of 54.15 cm (Table 6). When treated with Apron Star and transplanted into compost-treated plots, the Melka Zala variety showed the highest stand count percentage at harvest (85.42%), while the Mareko Fana variety in compost-untreated and control plots recorded the lowest stand count percentage (70.84%) (Table 6). There was significant variability in fruit length and diameter among hot pepper varieties treated with chemicals and transplanted into compost-treated plots compared to those transplanted into compost-untreated and control plots.

For instance, the longest fruit length (10.64 cm) was observed in the Mareko Fana variety when treated with Apron Star and transplanted into compost-treated plots, whereas the shortest fruit length (8.39 cm) was seen in the Melka Awaze variety when transplanted into compost-untreated and control plots (Table 6). Furthermore, the Melka Awaze variety treated with Apron Star and transplanted into compost-treated plots had the widest fruit diameter (1.73 cm) among all treatment combinations. Conversely, the Melka Zala variety treated with Cupricide and transplanted into compost-treated plots had the narrowest fruit diameter (1.53 cm). Interestingly, both the Mareko Fana and Melka Zala varieties treated with Apron Star and transplanted into compost-treated plots showed statistically nonsignificant differences (*p* ≤ 0.05) in terms of fruit diameter (Table 6).

#### 3.5.2. Yield Parameters

The research findings revealed that the Melka Zala variety had a higher number of fruits per plant compared to other treatment combinations. Specifically, Melka Zala variety seeds and seedlings treated with Apron Star and transplanted into compost-treated plots had the highest number of fruits per plant (148.43), whereas Melka Awaze variety had the lowest number of fruits per plant (101.21) when seedlings were transplanted into compost-untreated and control plots (Table 6). Significant differences (*p* ≤ 0.001) were observed in marketable and total fruit yield among hot pepper varieties treated with chemicals and transplanted into compost-treated and compost-untreated plots (Table 6). Notably, Melka Zala variety seeds and seedlings treated with Apron Star and transplanted into compost-treated plots achieved higher marketable fruit yield (2.42 t ha^−1^) and total fruit yield (2.47 t ha^−1^) compared to other treatment combinations (refer to Table 6).

The second higher marketable fruit yield (2.28 t ha^−1^) and total fruit yield (2.38 t ha^−1^) were achieved from the Melka Zala variety when its seeds and seedlings were treated with Cupricide and transplanted into compost-treated plots. Conversely, the Mareko Fana variety yielded lower marketable fruit (1.54 t ha^−1^) and total fruit (1.75 t ha^−1^) when seedlings were transplanted into compost-untreated and control plots (Table 6). The highest unmarketable fruit yield (0.23 t ha^−1^) came from Mareko Fana in compost-untreated control plots, while the lowest (0.05 t ha^−1^) was from Melka Zala treated with Apron Star and transplanted into compost-treated plots.

### 3.6. Relative Yield Loss

Significant differences in relative yield increase and loss were observed among various hot pepper varieties based on different treatment combinations (Table 7). The mean relative fruit yield losses were calculated for each treatment combination compared to the maximum protected plots and are presented in (Table 7). The control plots, without chemical seed and seedling treatments and untreated with compost, showed the highest grain yield losses: Mareko Fana at 13.90%, Melka Zala at 13.36%, and Melka Awaze at 8.33%, compared to the best performing plots of each variety where seeds and seedlings were treated with Apron Star and transplanted into compost-treated plots. Treating seeds and seedlings of Melka Awaze, Melka Zala, and Mareko Fana with Apron Star and transplanting them into compost-treated plots reduced relative yield loss by 1.96%, 3.64%, and 3.74%, respectively, compared to using Cupricide-treated seeds and seedlings in the same plots. Additionally, untreated (both chemical and compost) plots for each variety showed fruit yield losses ranging from 8.33% to 13.90% (Table 7).

### 3.7. Association of Hot Pepper Wilt Complex Disease and Yield Components

The coefficient of correlation is a measure of the level of association between dependent and independent variables. To this effect, variable relations were noted among disease and yield, and yield-related parameters and correlation coefficients are presented in (Table 8). The correlation matrix revealed that there were negative relationships between different magnitudes of disease parameters and growth, yield, and yield components, except for unmarketable fruit yield, which had a positive relationship with the disease parameters (Table 8). Thus, severity had a negative and significant correlation with SR (*r* = −0.77^∗∗∗^), PH (*r* = −0.55^∗∗∗^), SCH (*r* = −0.74^∗∗∗^), FD (*r* = −0.34^∗∗^), NFPP (*r* = −0.56^∗∗∗^), MFY (*r* = −0.72^∗∗∗^), and TFY (*r* = −0.67^∗∗∗^). Likewise, AUDPC values exhibited negative and highly significant (*p* ≤ 0.001) correlation with SR (*r* = −0.79^∗∗∗^), PH (*r* = −0.65^∗∗∗^), SCH (*r* = −0.80^∗∗∗^), FD (*r* = −0.42^∗∗^), NFPP (*r* = −0.46^∗∗∗^), MFY (*r* = −0.77^∗∗∗^), and TFY (*r* = −0.73^∗∗∗^) in the two cropping seasons. On the other hand, UMFY values were positively and highly correlated with severity (*r* = 0.77^∗∗∗^) and AUDPC (*r* = 0.79^∗∗∗^) (Table 8). Total fruit yield consistently had positively and highly significant (*p* ≤ 0.001) relationships with MFY (*r* = 0.99^∗∗∗^), SR (*r* = 0.73^∗∗∗^), NFPP (*r* = 0.61^∗∗∗^), and SCH (*r* = 0.40^∗∗∗^) in the cropping seasons. Similarly, severity was positively and highly and significantly correlated with AUDPC (*r* = 0.95^∗∗∗^).

## 4. Partial Budget Analysis

The partial budget analysis demonstrated that integrating compost, host resistance, and chemical treatments for seeds and seedlings resulted in the highest net benefit compared to compost-untreated and control plots.  Table 9 shows significant variations in net benefit and benefit–cost ratio among the different treatment combinations. Specifically, treating Melka Zala variety seeds and seedlings with Apron Star and transplanting them into compost-treated plots yielded the highest net benefit of $8294.20 ha^−1^ and a benefit–cost ratio (3.24). The second-highest net benefit of $7859.36 ha^−1^ was observed when Melka Zala seeds and seedlings were treated with Apron Star and transplanted into compost-untreated plots. Conversely, the lowest net benefit of $4143.89 ha^−1^ and a cost–benefit ratio (1.98) were calculated when Mareko Fana seedlings were transplanted into compost-untreated plots without any chemical treatment (Table 9). This indicates that the use of compost, host resistance, and fungicide treatments significantly influenced the economic outcomes of hot pepper cultivation.

Specifically, treating Melka Zala seeds and seedlings with Apron Star and transplanting them into compost-treated plots led to a 50.04% net benefit increase and a 38.89% benefit–cost ratio increase compared to Mareko Fana seeds and seedlings without chemical treatment and transplanted into compost-untreated plots. Likewise, using Apron Star on Melka Awaze seeds and seedlings and transplanting them into compost-treated plots led to a 33.96% net benefit increase and a 19.18% cost–benefit ratio increase compared to untreated Mareko Fana in compost-untreated plots. Overall, treating Melka Zala seeds and seedlings with Apron Star and transplanting them into compost-treated plots doubled the net benefit and marketable fruit yield compared to Mareko Fana seeds and seedlings without chemical treatment in compost-untreated plots.

## 5. Discussion

Wilt complex disease poses a severe threat to hot pepper (*Capsicum annuum* L.) production by significantly reducing both yield and quality. This disease complex, caused by a combination of soilborne pathogens including *Fusarium* spp., *Rhizoctonia* spp., and *Ralstonia solanacearum*, leads to plant wilting, chlorosis, and ultimately death, severely reducing crop productivity and marketable yield [19, 50]. The study found that combining host resistance, compost application, and chemical treatments significantly reduced hot pepper wilt disease severity, AUDPC, and disease progress while increasing fruit yield. Recent studies support these findings, indicating that single management methods are ineffective against the multipathogen hot pepper wilt complex [19]. The study showed that Apron Star treatments, when combined with compost application and different varieties, resulted in lower disease severity, AUDPC, disease progress curve, and disease progress rate compared to Cupricide and the control. This study aligns with Rather et al. [51], who found that an integrated approach involving seed and seedling treatment combined with fungicide foliar spraying was highly effective, reducing bell pepper wilt disease by 59.8% compared to the control. Lamichhane et al. [52] emphasized that managing damping-off effectively involves multiple strategies, including seed treatment, resistant cultivars, optimal cropping practices, and timely interventions with pesticides and biocontrol agents.

Gadhave et al. [53] discovered that systemic fungicides such as carbendazim, carboxin, thiophanate methyl, benomyl, and difenoconazole significantly hindered the growth of *F. oxysporum* f.sp. *lycopersici* under laboratory conditions. Similarly, Meyer and Hausbeck [54] observed that applying fungicides via drip lines or as soil directed sprays effectively suppressed Phytophthora crown and root rot diseases. Furthermore, Ram et al. [55] observed that treating seeds and seedlings with captan, metalaxyl, and carboxin could suppress wilt causing pathogens and other harmful microorganisms. The present study's findings are in line with those of Menge et al. [28], who demonstrated that combining fungicides, bioagents, and botanicals significantly reduced *Fusarium oxysporum* f.sp. *capsici* in pot culture experiments. This study highlights that chemical treatments for seeds and seedlings can reduce the pressure of hot pepper wilt complex disease. Specifically, Apron Star was more effective than Cupricide in treating hot pepper varieties, likely due to Apron Star's active ingredients, which include thiamethoxam 20%, metalaxyl 20%, and difenoconazole 2%. The systemic nature of metalaxyl and difenoconazole contributes to Apron Star's superior ability to control disease pressure compared to Cupricide.

The research illustrated that when hot pepper varieties were transplanted into compost-treated plots and treated with chemical seed and seedling treatments, they showed reduced disease severity, AUDPC, disease progress curve, and disease progress rate in comparison to plots that were untreated or served as controls. Compost enhances soil physical and chemical properties, promoting plant vigor and resilience to stresses. Additionally, compost increases the number and activity of microorganisms that suppress plant pathogenic fungi and bacteria. The results align with Kurabachew and Ayana [26], who found that soil amendments are effective in managing *R. solanacearum* and *Pythium*-related diseases in major solanaceous crop regions of Ethiopia. Similarly, Abada et al. [56] reported that using organic amendments like composted plant debris can enhance soil fertility, recycle agricultural waste, and effectively control soilborne plant diseases. Applying farmyard manure (FYM) or vermicompost to the soil notably decreased wilt and root rot and enhanced chili fruit yield compared to untreated conditions [57]. Previous research by Reijs et al. [27] demonstrated an 80% reduction in *Fusarium* wilt in chili plants through the use of organic soil amendments. Previous studies have confirmed that adding organic matter to soil improves its physical and chemical properties, benefiting crop growth and suppressing soilborne diseases like *Fusarium oxysporum* [58, 59]. Additionally, Zhai et al. [60] noted that organic residues not only provide nutrients and organic matter but also potentially increase the size, diversity, and activity of microbial populations in the soil. Recently, Rather et al. [51] reported that combining seed and seedling treatments with fungicide foliar sprays was the most effective method for managing bell pepper wilt complex disease, achieving a 59.8% reduction in disease compared to the control.

The study revealed that combining compost applications, host resistance, and chemical seed and seedling treatments significantly impacted the growth and yield of hot pepper varieties. Earlier research by Cianchetta and Davis [61] and Abd-Elgawad and Askary [62] indicated that host genetics, soil amendments, and fertilizers are essential components of integrated pest management. Seedling survival percentage and stand count at harvest were higher in plots treated with chemicals compared to untreated plots. This is likely due to damping-off disease, caused by *Rhizoctonia solani* and *Pythium* spp., which affect seedlings early and are characterized by stem base rot. Conversely, chemical-treated plots made the seedlings more vigorous and better able to withstand disease pressure.

Regardless of treatment combination effects, the Melka Zala variety had the highest marketable and total fruit yield. This suggests that the observed differences in agronomic traits were influenced not only by disease pressure and treatment effects but also by the genetic makeup of the hot pepper varieties. Getahun and Habtie [6] previously reported that Melka Zala and Melka Awaze had superior fruit yields due to their tolerance to disease and excellent vegetative growth, including height, which contributed to their outstanding yield performance. However, the current study contradicts previous research by Gebremeskel et al. *[*63*]*, which found that the variety Melka Awaze had a higher yield than Melka Zala under irrigation in Raya Valley, Tigray, Ethiopia.

Melka Zala seeds and seedlings treated with Apron Star and transplanted into compost-treated plots exhibited higher yields. This combination, along with the genetic potential of the variety, likely helped suppress the development of wilt complex disease. Correlation analysis revealed a significant positive correlation among disease parameters, while growth, yield, and yield components showed a strong negative correlation with disease parameters. The significant relationship between disease severity and AUDPC indicates that the factors contributing to the hot pepper wilt complex disease and yield reduction were complementary and linearly associated [44]. Furthermore, the negative correlation between disease parameters and fruit yield suggests that the wilt disease significantly contributed to yield loss in hot pepper by adversely affecting yield components such as fruit number per plant, fruit length, and diameter. The disease also compromised the root system, leading to leaf withering, stem collapse, and damage to the vascular system. Menge et al. [28] reported that *Fusarium* wilt causes plant wilting, upward and inward leaf rolling, yellowing, and eventually plant death. The previous study conducted by Sapkota et al. [64] demonstrated that hot pepper fields infested with wilt pathogens experienced yield reductions of up to 50% compared to healthy fields. In the present study, the combined application of wilt complex disease management strategies significantly increased the net benefit and marketable fruit yield compared to untreated and control plots. Additionally, these methods played a crucial role in reducing the overall yield loss of hot pepper.

## 6. Conclusion

Hot pepper wilt complex disease has recently become a significant challenge in the study area's hot pepper production. In the study, Melka Zala variety seeds and seedlings treated with Apron Star and transplanted into compost-treated plots showed reduced disease severity, lower AUDPC values, a slower disease progress curve, and a reduced disease progress rate compared to other treatment combinations. These treatments also resulted in higher fruit yields. Conversely, the Mareko Fana variety exhibited significantly higher disease severity, higher AUDPC values, a faster disease progress curve, and a higher disease progress rate, along with lower total fruit yields when its seeds and seedlings were not treated with chemicals and were transplanted into compost-untreated plots. Moreover, treating Melka Zala seeds and seedlings with Apron Star fungicide and transplanting them into compost-treated plots resulted in twice the net benefit compared to untreated Mareko Fana seeds and seedlings in compost-untreated plots. The best outcomes were achieved with Melka Zala variety seeds and seedlings treated with Apron Star and transplanted into compost-treated plots. Thus, an integrated disease management strategy is recommended for controlling hot pepper wilt complex disease in the study area and could be vital for similar agroecological regions in the country. Future research should focus on conducting long-term field trials to validate the effectiveness of these integrated management strategies across different agroecological zones.

## Figures and Tables

**Table 1:** Characteristic features of chemicals tested for the management of hot pepper wilt complex disease.

**Common (trade name)**	**Active ingredient**	**Product formulation**	**Target crop**	**Target disease**	**Application rate**
Cupricide 77% WP	Copper hydroxide 77% *W*/*W*	Wettable powder	Tomato and other vegetable crops	Bacterial spot and fungal foliar diseases	2.0 kg ha^−1^ seed with a dilution water volume of 200 L
Apron Star 42% WS	Thiamethoxam 20% + metalaxyl 20% + difenoconazole 2%	Wettable substrate	Cereal, pulse, and vegetable	Soilborne diseases and seedling blight	2.0 g kg^−1^ seed with a dilution water volume of 400 L

*Note: Source:* Data were sourced and organized from MoA [37, 38] and product's package booklet.

**Table 2:** Characteristic features of hot pepper varieties used for the study at Geraye FTC in Jabi Tehena District, northwestern Ethiopia, during the 2020 and 2021 main rainy seasons.

**Genotype**	**Pedigree**	**Year of release**	**Days to maturity**	**Yield (t ha** ^**−1**^ **) on a research station**
Melka Awaze	PBC600	2006	100	2.0–2.8
Mareko Fana	NA	1976	120–135	1.5–2.5
Melka Zala	PBC972	2004	135	2.0–2.5

Abbreviation: NA, not available.

**Table 3:** Combined analysis of mean square values for utilizing compost applications, host resistance, and chemical treatments (for seeds and seedlings) to manage hot pepper wilt complex disease at Geraye FTC in the Jabi Tehena District of northwestern Ethiopia during the main rainy seasons of 2020 and 2021.

**Source of variation**	**d** **f**	**Disease parameter** ^**a**^		**Agronomic parameters**								
		**PSI**_**f**_	**AUDPC**	**SSP**	**PH**	**NFPP**	**FL**	**SCH**	**FD**	**MFY**	**UMFY**	**TFY**
Y	1	56.33⁣^∗∗∗^	416889.82⁣^∗∗∗^	86.85⁣^∗∗∗^	81.68⁣^∗∗∗^	1845.86⁣^∗∗∗^	0.18^ns^	13.00⁣^∗^	0.01⁣^∗∗^	0.04⁣^∗∗∗^	0.00⁣^∗^	0.02⁣^∗∗∗^
R(Y)	4	1.78^ns^	1606.48⁣^∗^	5.78^ns^	7.71^ns^	0.54^ns^	0.03^ns^	1.27^ns^	0.00⁣^∗^	0.00^ns^	0.00⁣^∗^	0.00^ns^
C	1	1134.26⁣^∗∗∗^	1130578.70⁣^∗∗∗^	336.48⁣^∗∗∗^	1167.69⁣^∗∗∗^	301.77⁣^∗∗∗^	1.75⁣^∗∗∗^	29.29⁣^∗∗^	0.01⁣^∗^	0.09⁣^∗∗∗^	0.04⁣^∗∗∗^	0.01⁣^∗∗∗^
Y⁣^∗^C	1	0.03^ns^	25208.33⁣^∗∗∗^	2.27^ns^	1.76^ns^	2.04^ns^	0.00^ns^	17.71⁣^∗^	0.00^ns^	0.00⁣^∗∗^	0.00⁣^∗∗^	0.00⁣^∗∗^
V	2	595.11⁣^∗∗∗^	273562.04⁣^∗∗∗^	414.11⁣^∗∗∗^	692.86⁣^∗∗∗^	9689.83⁣^∗∗∗^	9.15⁣^∗∗∗^	46.21⁣^∗∗∗^	0.01⁣^∗∗^	3.26⁣^∗∗∗^	0.04⁣^∗∗∗^	2.91⁣^∗∗∗^
Y⁣^∗^V	2	1.33^ns^	8562.04⁣^∗∗∗^	2.80^ns^	30.01⁣^∗∗∗^	911.53⁣^∗∗∗^	0.19^ns^	36.09⁣^∗∗∗^	0.00^ns^	0.00^ns^	0.00^ns^	0.00^ns^
C⁣^∗^V	2	1.04^ns^	15628.70⁣^∗∗∗^	24.50⁣^∗∗^	78.54⁣^∗∗∗^	26.19⁣^∗∗∗^	0.33^ns^	6.78^ns^	0.00^ns^	0.00^ns^	0.01⁣^∗^	0.00⁣^∗^
Y⁣^∗^C⁣^∗^V	2	1.04^ns^	1258.33^ns^	0.63^ns^	12.13⁣^∗^	2.53^ns^	0.14^ns^	0.63^ns^	0.04⁣^∗∗∗^	0.00^ns^	0.00^ns^	0.00^ns^
Chem	2	1596.00⁣^∗∗∗^	1011448.15⁣^∗∗∗^	158.99⁣^∗∗∗^	261.29⁣^∗∗∗^	111.42⁣^∗∗∗^	7.25⁣^∗∗∗^	138.45⁣^∗∗∗^	0.00⁣^∗∗∗^	0.04⁣^∗∗∗^	0.00⁣^∗∗^	0.00⁣^∗∗∗^
Y⁣^∗^ Chem	2	1.33^ns^	5137.04⁣^∗∗∗^	2.81^ns^	6.28^ns^	0.16^ns^	0.27^ns^	36.07⁣^∗∗∗^	0.03⁣^∗∗∗^	0.00⁣^∗^	0.00^ns^	0.00^ns^
C⁣^∗^ Chem	2	2.97.93⁣^∗∗∗^	65403.70⁣^∗∗∗^	26.13⁣^∗∗^	32.43⁣^∗∗∗^	5.56⁣^∗∗^	0.29^ns^	2.44^ns^	0.00^ns^	0.00^ns^	0.01⁣^∗∗∗^	0.00^ns^
Y⁣^∗^C⁣^∗^ Chem	2	6.37⁣^∗^	5511.11⁣^∗∗∗^	5.51^ns^	2.65^ns^	0.93^ns^	0.09^ns^	0.63^ns^	0.03⁣^∗∗∗^	0.00^ns^	0.00^ns^	0.00^ns^
V⁣^∗^ Chem	4	4.11⁣^∗^	2025.93⁣^∗^	7.69^ns^	24.12⁣^∗∗∗^	7.53⁣^∗∗∗^	1.93⁣^∗∗∗^	0.22^ns^	0.00^ns^	0.00^ns^	0.00^ns^	0.00^ns^
Y⁣^∗^V⁣^∗^ Chem	4	2.33^ns^	1050.93^ns^	6.60^ns^	8.77⁣^∗^	3.68⁣^∗∗^	0.03^ns^	2.58^ns^	0.00^ns^	0.00^ns^	0.00^ns^	0.00^ns^
C⁣^∗^V⁣^∗^ Chem	4	12.04⁣^∗∗∗^	18278.70⁣^∗∗∗^	21.78⁣^∗∗∗^	28.76⁣^∗∗∗^	4.66⁣^∗∗^	0.35⁣^∗^	9.63⁣^∗^	0.00^ns^	0.00^ns^	0.00^ns^	0.00⁣^∗^
Y⁣^∗^C⁣^∗^V⁣^∗^Chem	4	2.70^ns^	1577.78^ns^	7.14^ns^	1.55^ns^	1.07^ns^	0.11^ns^	3.49^ns^	0.00^ns^	0.00^ns^	0.00^ns^	0.00^ns^
Error	68	1.54	663.34	3.68	3.68	0.93	0.12	3.37	0.00	0.00	0.00	0.00
Mean	—	34.65	953.24	81.63	69.00	122.47	9.45	78.36	1.59	1.86	0.13	1.99
CV (%)	—	4.17	2.70	2.35	1.92	0.96	3.65	2.34	2.15	1.13	12.87	1.38

*Note:*⁣^∗^, ⁣^∗∗^, and ⁣^∗∗∗^ in each parameter refer to treatments that were significant, highly significant, and very highly significant, respectively.

Abbreviations: AUDPC, area under disease progress curve; C, compost; Chem, chemical; CV, coefficient of variation; FD, fruit diameter; FL, fruit length; MFY, marketable fruit yield; NFPP, number of fruits per plant; PH, plant height; R, replication; SCH, stand count at harvest; SSP, seedling survival percentage; TFY, total fruit yield; UMFY, unmarketable fruit yield; V, variety; Y, year.

^a^PSI_f_, final percent severity index, which was assessed at 85 days after transplanting.

**Table 4:** The interaction effect of compost applications, host resistance, and chemical treatments on hot pepper wilt complex disease severity and the area under the disease progress curve at Geraye FTC in the Jabi Tehena District of northwestern Ethiopia during the main rainy seasons of 2020 and 2021.

**Compost**	**Variety**	**Chemical**	**PSI**_**i**_	**PSI**_**f**_	**AUDPC (%-days)**
Compost-treated	Melka Awaze	Apron Star	9.33^j^	26.33^h^	615.00^k^
		Cupricide	14.00^f–i^	31.67^f^	808.33^i^
		Control	16.00^cde^	36.33^e^	1045.00^d^
	Mareko Fana	Apron Star	13.00^hi^	30.33^fg^	823.33^hi^
		Cupricide	13.33^hi^	36.00^e^	963.33^f^
		Control	14.33^fgh^	38.33^d^	1046.67^d^
	Melka Zala	Apron Star	6.22^k^	23.00^i^	478.33^l^
		Cupricide	11.67^i^	27.33^h^	700.00^j^
		Control	15.00^efg^	31.00^fg^	816.67^i^

Compost-untreated	Melka Awaze	Apron Star	14.33^fgh^	31.00^fg^	860.00^gh^
		Cupricide	17.33^abc^	35.33^e^	1013.33^ed^
		Control	17.67^abc^	47.67^b^	1223.33^b^
	Mareko Fana	Apron Star	15.33^efg^	41.00^c^	973.33^ef^
		Cupricide	16.67^bcd^	34.67^e^	1055.00^d^
		Control	18.67^a^	54.00^a^	1426.67^a^
	Melka Zala	Apron Star	7.33^k^	27.00^h^	678.33^j^
		Cupricide	13.72^ghi^	29.67^g^	896.67^g^
		Control	16.33^b–e^	43.00^c^	1101.67^c^

CV (%)			9.94	4.43	4.01

LSD (0.05)			1.59	1.75	42.26

*p* value			< 0.0001	< 0.0001	< 0.0001

*Note:* PSI_i_ and PSI_f_ refer to the initial and final percent severity index of hot pepper wilt complex disease, which were assessed at 45 and 85 days after seedling transplanting in both seasons. Letters in each column refer to means with the same letter in the same column that are not significantly different.

**Table 5:** Interaction effect of compost application, host resistance, and chemical treatments on disease progress rate (units day^−1^) of hot pepper wilt complex disease in Jabi Tehena District of northwestern Ethiopia during the 2020 and 2021 main rainy seasons.

**Compost**	**Variety**	**Chemical**	**DPR** ^**a**^	**Intercept**	**SE of intercept** ^**b**^	**SE of rate** ^**b**^	**R** ^2^ ** (%)** ^**c**^
Compost-treated	Melka Awaze	Apron Star	0.0056	−0.1503	0.0569	0.0009	76.99
		Cupricide	0.0059	−0.1262	0.0579	0.0012	64.09
		Control	0.0072	−0.1810	0.0661	0.0009	80.22
	Mareko Fana	Apron Star	0.0053	−0.1477	0.0707	0.0011	66.01
		Cupricide	0.0059	−0.1530	0.1130	0.0017	47.79
		Control	0.0062	−0.1670	0.0672	0.0009	75.87
	Melka Zala	Apron Star	0.0034	−0.1172	0.0732	0.0011	60.71
		Cupricide	0.0058	−0.1612	0.0478	0.0009	73.00
		Control	0.0072	−0.1701	0.0623	0.0009	82.00

Compost-untreated	Melka Awaze	Apron Star	0.0053	−0.0859	0.0305	0.0005	91.08
		Cupricide	0.0080	−0.1992	0.0530	0.0008	88.69
		Control	0.0091	−0.4010	0.1910	0.0029	55.05
	Mareko Fana	Apron Star	0.0082	−0.2048	0.0844	0.0013	76.32
		Cupricide	0.0064	−0.1512	0.0550	0.0008	82.36
		Control	0.0114	−0.3290	0.1360	0.0020	69.70
	Melka Zala	Apron Star	0.0067	−0.1370	0.0861	0.0013	67.52
		Cupricide	0.0080	−0.2310	0.1210	0.0018	60.08
		Control	0.0089	−0.2680	0.0193	0.0029	42.25

^a^DPR = disease progress rate obtained from the regression line of severity (percent) against a time of disease assessment (days).

^b^SE = standard error of rate and parameter estimate (intercept).

^c^ *R* ^2^ = coefficient of determination for the monomolecular epidemiological model.

**Table 6:** Interaction effects of compost applications, host resistance, and chemical treatments on yield and yield components of hot pepper during the 2020 and 2021 main rainy seasons in Geraye FTC, Jabi Tehena District, northwestern Ethiopia.

**Compost**	**Variety**	**Chemical**	**Yield and yield components**								
			**SSP (%)**	**PH (cm)**	**NFPP**	**SCH**	**FL (cm)**	**FD (cm)**	**MFY (t ha** ^**−1**^ **)**	**UMFY (t ha** ^**−1**^ **)**	**TFY (t ha** ^**−1**^ **)**
Compost-treated	Melka Awaze	Apron Star	85.94^b^	82.97^a^	111.20^l^	81.77^bc^	10.06^b^	1.73^a^	1.97^f^	0.07^jk^	2.04^d^
		Cupricide	82.82^cd^	74.98^b^	108.17^m^	79.69^d^	8.47^ji^	1.59^b–e^	1.90^g^	0.10^hi^	2.00^e^
		Control	79.69^efg^	72.63^c^	107.65^m^	77.09^fgh^	8.96^gh^	1.56^ef^	1.86^h^	0.11^gh^	1.97^ef^
	Mareko Fana	Apron Star	82.29^d^	70.42^e^	119.07^g^	79.17^de^	10.80^a^	1.64^b^	1.74^j^	0.13^ef^	1.87^j^
		Cupricide	79.17^fg^	68.82^f^	117.48^h^	75.52^hij^	9.21^fg^	1.59^de^	1.64^l^	0.16^bcd^	1.80^k^
		Control	78.13^gh^	65.70^gh^	115.35^i^	73.96^jkl^	9.94^bc^	1.56^ef^	1.58^m^	0.17^bc^	1.75^l^
	Melka Zala	Apron Star	90.63^a^	75.38^b^	148.43^a^	85.42^a^	9.61^cde^	1.64^b^	2.42^a^	0.05^k^	2.47^a^
		Cupricide	86.98^b^	69.66^efg^	145.00^b^	82.29^b^	9.71^bcd^	1.53^f^	2.28^b^	0.10^jik^	2.38^b^
		Control	84.90^bc^	65.43^h^	137.03^e^	78.65^def^	9.48^def^	1.53^f^	2.22^c^	0.11^gh^	2.33^c^

Compost-untreated	Melka Awaze	Apron Star	83.34^cd^	71.98^cd^	104.80^n^	76.57^gh^	9.01^gh^	1.59^cde^	1.87^jh^	0.13^de^	2.00^h^
		Cupricide	77.61^gh^	71.03^de^	103.42^o^	76.04^ghi^	8.82^hi^	1.62^bcd^	1.81^i^	0.14^efg^	1.95^i^
		Control	81.77^de^	66.86^g^	101.21^p^	73.44^kl^	8.39^j^	1.56^ef^	1.70^k^	0.16^b^	1.87^j^
	Mareko Fana	Apron Star	79.17^fg^	60.88^j^	114.10^j^	74.48^jik^	10.64^a^	1.57^def^	1.64^l^	0.15^c–f^	1.79^k^
		Cupricide	76.57^h^	57.82^k^	113.90^j^	72.40^lm^	9.12^fgh^	1.59^de^	1.58^m^	0.17^b^	1.74^l^
		Control	73.44^i^	54.15^l^	112.38^k^	70.84^m^	10.05^b^	1.58^de^	1.39^n^	0.23^a^	1.61^m^
	Melka Zala	Apron Star	82.82^cd^	65.00^h^	142.27^c^	80.21^cd^	9.45^def^	1.60^b–e^	2.22^c^	0.06^k^	2.30^d^
		Cupricide	81.25^def^	62.75^i^	139.82^d^	77.61^efg^	9.31^efg^	1.59^de^	2.13^d^	0.13^fg^	2.26^e^
		Control	82.82^cd^	62.20^i^	133.10^a^	75.52^hij^	9.17^fgh^	1.56^ef^	2.00^e^	0.14^def^	2.14^f^

Mean			81.63	67.63	120.79	77.25	9.45	1.59	1.88	0.13	2.01

CV (%)			2.71	1.55	0.67	2.22	3.58	2.55	1.34	14.46	1.34

*p* value			< 0.0001	< 0.0001	< 0.0001	< 0.0001	< 0.0001	< 0.0001	< 0.0001	< 0.0001	< 0.0001

*Note:* Letters in each column refer to means with the same letter in the same column that are not significantly different.

Abbreviations: AUDPC, area under disease progress curve; FD, fruit diameter; FL, fruit length; MFY, marketable fruit yield; NFPP, number of fruits per plant; PH, plant height; PSI, percent severity index; SCH, stand count at harvest; SSP, seedling survival percentage; TFY, total fruit yield; UMFY, unmarketable fruit yield.

**Table 7:** The interaction effect of compost applications, host resistance, and chemical treatments on total dry fruit yield, relative yield, and relative yield loss of hot pepper in the Jabi Tehena District, northwestern Ethiopia, during the 2020 and 2021 main rainy seasons.

**Variety**	**Compost**	**Chemical**	**Yield parameters**		
			**TFY**	**RY (%)**	**RYL (%)**
Melka Awaze	Compost-treated	Apron Star	2.04	100	0.00
		Cupricide	2.00	98.04	1.96
		Control	1.97	96.57	3.43
	Compost-untreated	Apron Star	2.00	98.04	2.00
		Cupricide	1.95	95.59	4.41
		Control	1.87	91.67	8.33

Mareko Fana	Compost-treated	Apron Star	1.87	100	0.00
		Cupricide	1.80	96.23	3.74
		Control	1.75	93.58	6.42
	Compost-untreated	Apron Star	1.79	95.72	4.44
		Cupricide	1.74	93.05	6.95
		Control	1.61	86.09	13.90

Melka Zala	Compost-treated	Apron Star	2.47	100	0.00
		Cupricide	2.38	96.36	3.64
		Control	2.33	94.33	5.67
	Compost-untreated	Apron Star	2.30	93.12	6.88
		Cupricide	2.26	91.45	8.50
		Control	2.14	86.64	13.36

Abbreviations: RY (%), relative yield; RYL (%), relative yield loss; TFY, total fruit yield.

**Table 8:** The coefficients of correlation (*r*) among growth yield and yield components of hot pepper and wilt complex disease parameters at Geraye FTC in Jabi Tehena District, northwestern Ethiopia, during the 2020 and 2021 main rainy seasons.

**Parameter**	**SSP**	**PH (cm)**	**NFPP**	**SCH**	**FL (cm)**	**FD (cm)**	**MFY (t ha** ^**−1**^ **)**	**UMFY (t ha** ^**−1**^ **)**	**TFY (t ha** ^**−1**^ **)**	**PSI**	**AUDPC**
SR	1	0.55⁣^∗∗∗^	0.51⁣^∗∗∗^	0.75⁣^∗∗∗^	0.05^ns^	0.25⁣^∗∗^	0.74⁣^∗∗∗^	−0.69⁣^∗∗∗^	0.73⁣^∗∗∗^	−0.77⁣^∗∗∗^	−0.79⁣^∗∗∗^
PH		1	−0.12^ns^	0.64⁣^∗∗∗^	−0.14^ns^	0.43⁣^∗∗∗^	0.27⁣^∗∗∗^	−0.59⁣^∗∗∗^	0.19⁣^∗^	−0.55⁣^∗∗∗^	−0.65⁣^∗∗∗^
NFPP			1	0.53⁣^∗∗∗^	0.21⁣^∗^	−0.05^ns^	0.57⁣^∗∗∗^	−0.19⁣^∗^	0.61⁣^∗∗∗^	−0.56⁣^∗∗∗^	−0.46⁣^∗∗∗^
SCH				1	−0.01^ns^	0.25⁣^∗∗^	0.43⁣^∗∗∗^	−0.46⁣^∗∗∗^	0.40⁣^∗∗∗^	−0.74⁣^∗∗∗^	−0.80⁣^∗∗∗^
FL					1	0.19⁣^∗^	−0.10^ns^	−0.01^ns^	−0.11^ns^	−0.19^ns^	−0.09^ns^
FD						1	0.08^ns^	−0.31⁣^∗∗^	0.04^ns^	−0.34⁣^∗∗∗^	−0.42⁣^∗∗∗^
MFY							1	−0.77⁣^∗∗∗^	0.99⁣^∗∗∗^	−0.72⁣^∗∗∗^	−0.77⁣^∗∗∗^
UMFY								1	−0.68⁣^∗∗∗^	0.79⁣^∗∗∗^	0.77⁣^∗∗∗^
TFY									1	−0.83⁣^∗∗∗^	−0.73⁣^∗∗∗^
PSI										1	0.95⁣^∗∗∗^
AUDPC											1

Abbreviations: AUDPC, area under disease progress curve; FD, fruit diameter; FL, fruit length; MFY, marketable fruit yield; NFPP, number of fruits per plant; ns, nonsignificant; PH, plant height; PSI, percent severity index; SCH, stand count at harvest; SSP, seedling survival percentage; TFY, total fruit yield; UMFY, unmarketable fruit yield.

⁣^∗^Significant at *p* < 0.05. ⁣^∗∗^Highly significant at *p* < 0.01. ⁣^∗∗∗^Very highly significant at *p* < 0.001.

**Table 9:** Partial budget analysis of integrated management strategies for hot pepper wilt complex disease using compost application, host resistance, and chemical treatment in Geraye FTC, Jabi Tehena District, during the 2020 and 2021 main rainy seasons.

**Compost**	**Varity**	**Fungicide**	**Cost benefit variables**					
			**MFY (kg ha** ^**−1**^ **)**	**AFY (kg ha** ^**−1**^ **) 10% down**	**GB ($ ha** ^**−1**^ **)**	**TPC ($ ha** ^**−1**^ **)**	**NB ($ ha** ^**−1**^ **)**	**BCR**
Compost-treated	Melka Awaze	Apron Star	1970	1773	8837.95	2562.56	6275.38	2.45
		Cupricide	1900	1710	8523.91	2557.47	5966.43	2.33
		Control	1860	1674	8344.46	2554.42	5790.03	2.27
	Mareko Fana	Apron Star	1740	1566	7806.10	2562.56	5243.54	2.05
		Cupricide	1640	1476	7357.48	2557.47	4800.00	1.88
		Control	1580	1422	7088.30	2554.42	4533.88	1.77
	Melka Zala	Apron Star	2420	2178	10856.77	2562.56	8294.20	3.24
		Cupricide	2280	2052	10228.69	2557.47	7671.21	3.00
		Control	2220	1998	9959.51	2554.42	7405.09	2.90

Compost-untreated	Melka Awaze	Apron Star	1870	1683	8389.32	2100.15	6289.16	2.99
		Cupricide	1810	1629	8120.14	2095.07	6025.07	2.88
		Control	1700	1530	7626.65	2092.02	5534.64	2.65
	Mareko Fana	Apron Star	1640	1476	7357.48	2100.15	5257.32	2.50
		Cupricide	1580	1422	7088.30	2095.07	4993.23	2.38
		Control	1390	1251	6235.91	2092.02	4143.89	1.98
	Melka Zala	Apron Star	2220	1998	9959.51	2100.15	7859.36	3.74
		Cupricide	2130	1917	9555.75	2095.07	7460.68	3.56
		Control	2000	1800	8972.53	2092.02	6880.52	3.29

Abbreviations: $, symbol of USD; AFY, adjusted fruit yield; BCR, benefit–cost ratio; GB, gross benefit; MFY, marketable fruit yield; NB, net benefit; TPC, total production cost.

## Data Availability

Data will be available if required by editors and reviewers.
